# Spin and orbital structure of the first six holes in a silicon metal-oxide-semiconductor quantum dot

**DOI:** 10.1038/s41467-018-05700-9

**Published:** 2018-08-14

**Authors:** S. D. Liles, R. Li, C. H. Yang, F. E. Hudson, M. Veldhorst, A. S. Dzurak, A. R. Hamilton

**Affiliations:** 1 School of Physics, University of New South Wales, Sydney, NSW 2052 Australia; 2 QuTech and Kavli Institute of Nanoscience, TU Delft, 2600 GA Delft, The Netherlands; 3 Centre for Quantum Computation and Communication Technology, School of Electrical Engineering and Telecommunications, The University of New South Wales, Sydney, NSW 2052 Australia

## Abstract

Valence band holes confined in silicon quantum dots are attracting significant attention for use as spin qubits. However, experimental studies of single-hole spins have been hindered by challenges in fabrication and stability of devices capable of confining a single hole. To fully utilize hole spins as qubits, it is crucial to have a detailed understanding of the spin and orbital states. Here we show a planar silicon metal-oxide-semiconductor-based quantum dot device and demonstrate operation down to the last hole. Magneto-spectroscopy studies show magic number shell filling consistent with the Fock–Darwin states of a circular two-dimensional quantum dot, with the spin filling sequence of the first six holes consistent with Hund’s rule. Next, we use pulse-bias spectroscopy to determine that the orbital spectrum is heavily influenced by the strong hole–hole interactions. These results provide a path towards scalable silicon hole-spin qubits.

## Abstract (web summary)

For solid state qubits, silicon MOS structures offer great scalability, while hole spins promise high performance qubit operation. Liles et al. have combined these two features in a planar silicon quantum dot device that operates as an artificial atom down to the single-hole limit.

## Introduction

The spin states of electrons confined in semiconductor quantum dots form a promising platform for quantum computation^1–3^. Recent studies of silicon complementary metal-oxide-semiconductor (CMOS) qubits have shown coherent manipulation of electron spin states with extremely high fidelity^4^. However, manipulation of single electron spins requires large oscillatory magnetic fields to be generated on-chip, making it difficult to address individual qubits when scaling up to multi-qubit devices^4,5^. In addition, electron spins experience a strong hyperfine coupling to the nuclei spin of the host crystal, which limits spin coherence times^6^. While coherence time can be significantly improved by using isotopically purified material systems such as silicon^4^, the isolation of electron spins makes it difficult to perform fast operations, thereby sacrificing operation speed for coherence time.

Hole spins in semiconductor quantum dots are attracting significant attention as candidates for fast, highly coherent spin qubits^7,8^. These two key factors for qubit operation, coherence and speed, can be enhanced due to the unique spin properties of valence band holes. Hole spins are predicted to have long coherence times due to the weak hyperfine coupling to nuclear spins^9,10^. Optical studies of hole quantum dots suggest a 10 to 100 times enhancement of ${T}_2^ \ast$ over electron spins^11,12^. By significantly suppressing a leading source of decoherence, *p*-type materials may provide highly coherent spin qubit systems. Hole-spin-based qubits have been demonstrated to have rapid operation times^13,14^, due to the inherently strong spin–orbit coupling, which also allows spin states to be controlled locally with electric fields applied to gate electrodes^15–18^. Hole spins therefore provide a highly scalable system of easily addressable, fast qubits.

Despite these promising properties, hole-based quantum dots still face technological challenges that have been overcome in electron systems more than a decade ago^3^. Planar silicon CMOS quantum dots are amongst the most promising semiconductor system to implement spin qubits. This type of device is suitable for high-frequency spin manipulation experiments^18^, and is optimized for scalability up to many qubits^19,20^. However, creating a planar silicon CMOS quantum dot capable of confining a single hole has been a challenge^21–24^. Previous studies of planar silicon-based hole quantum dots have used transport measurements to study the addition spectrum of the quantum dot^21–23^. However, as these devices approach the few hole regime, the tunnel barriers become extremely opaque, and the transport signal falls precipitously. This has hampered studies of hole quantum dots containing one and two holes, which is the most widely used regime for spin-based quantum computation applications.

In this work, we present experimental observations of the first six hole states in a surface-gated silicon metal-oxide-semiconductor quantum dot. Confirmation that the device can reach the single-hole regime is possible since the device incorporates a charge sensor. More specifically, we characterize the spin filling sequence and orbital structure of the first six holes. In particular, we find the spin filling consistent with the Fock–Darwin states, and demonstrate that the hole–hole interaction energy is 90% of the orbital energy. These results are a promising step towards hole-based spin qubits, since we present a stable single-hole quantum dot operating in the same planar geometry that has already proven highly successful for electron spin qubits^4,18,25^. In addition, silicon metal-oxide-semiconductor (MOS) technology has the advantages of compatibility with current industrial technology, allowing potential for scale-up to multiple highly coherent fast qubits.

## Results

### Operation down to the last hole

**Fig. 1:**
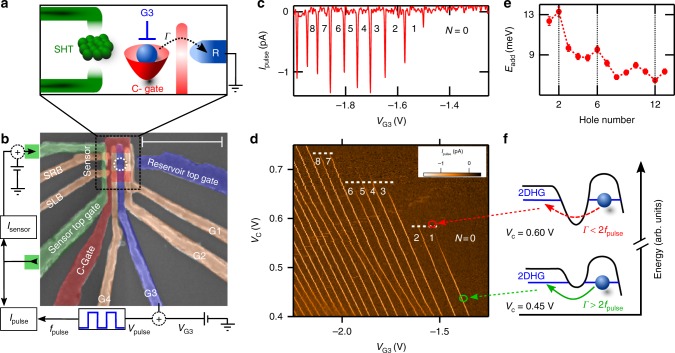
Silicon quantum dot with a charge sensor, capable of reaching the last hole. **a** Schematic of the device concept. The device consists of a quantum dot coupled to a single reservoir (R), with an adjacent single-hole transistor (SHT) charge sensor. The tunnel rate between the dot and reservoir (*Γ*) can be tuned using the C-gate voltage (*V*_C_), and the dot occupation can be controlled with G3-gate voltage (*V*_G3_). **b** False-colored scanning electron microscope image of an identical device, with the measurement schematic. The scale bar is 500 nm. **c** Depletion of the last 10 holes in the quantum dot, showing the *V*_pulse_ induced signal on the charge sensor measured at *V*_C_ = 0.47 V. **d** Charge stability diagram, showing the number of holes on the dot as a function of the confining gate (C-gate) and pulse gate (G3) potentials. The horizontal white lines highlight the disappearance of the charge transition signals in distinct groupings, indicating shell filling. Measurements performed for *V*_pulse_ = 3 mV and *f*_pulse_ = 333 Hz. A slight bending in the lines in the vicinity of *V*_G3_ = −2 V, *V*_C_ = 0.55 V is due to coupling to nearby confined charge. **e** The hole addition energy extracted from **d**, showing peaks at *N* = 2 and 6 consistent with shell filling. The error bars result from the standard error in the mean of 20 measurements over a range of *V*_C_. **f** Schematic diagram depicting the change in the tunnel barrier height between the *V*_C_ and *V*_G3_ configurations indicated by the green and red circle in **d**. The tunnel rate, *Γ*, between the dot and the 2D hole gas (2DHG) is influenced by changing *V*_C_, causing the charge transition signals to disappear when the tunnel time becomes comparable to the period of the pulses applied to G3. See Methods section for additional experimental parameters

Figure 1a shows a schematic of the device, and Fig. 1b shows a scanning electron microscope image of a nominally identical device to the one used in this study. This device features a planar hole quantum dot connected to a single reservoir (R) of two-dimensional (2D) holes. The number of holes on the dot *N* is controlled with the bias on gate G3, and the charge occupation in the dot is monitored using an adjacent charge sensor (single-hole transistor (SHT)). The dot to reservoir tunnel rate *Γ* can be tuned without affecting the dot confinement shape using the bias applied to the C-gate. By measuring the charge occupation with a charge sensor we are able to study hole states even when the tunnel rate between the dot and reservoir is much smaller than can be detected in transport.

In order to characterize the addition spectrum of holes in the quantum dot, we employ a pulse-bias technique^26^, which allows the charge occupation of the dot to be monitored using an adjacent SHT charge sensor. We apply a 1 mV DC excitation to the SHT’s source ohmic contact (top green square in Fig. 1b), and add a continuous square wave of magnitude *V*_pulse_ and frequency *f*_pulse_ to gate G3. The modulation of the DC sensor current by *V*_pulse_, called *I*_pulse_, is sensitive to d*Q*_dot_/d*V*_G3_ (as long as *Γ* > 2*f*_pulse_). In Fig. 1c we show a measurement of *I*_pulse_ as *V*_G3_ is swept. At specific values of *V*_G3_ a hole is able to tunnel off and on the dot during the positive/negative phase of *V*_pulse_. This charge movement decreases the modulation on the DC sensor current, causing a negative spike in *I*_pulse_ of width *V*_pulse_ in the *V*_G3_ scan. The measurement of Fig. 1c was repeated over a range of *V*_C_ to produce the charge stability diagram in Fig. 1d. The identification of the last hole in the dot is confirmed by the absence of any additional charge transitions beyond the region labeled *N* = 0 in Fig. 1d.

The spacing of the charge transition lines in Fig. 1d provides clear evidence for orbital shell filling of the hole quantum dot^27^. We extracted the addition energy (*E*_add_(*N*) = *μ*_*N*+1_ − *μ*_*N*_) by measuring the spacing Δ*V*_G3_ between consecutive Coulomb peaks, then converted Δ*V*_G3_ to energy using the lever arm of 0.17 eV/V  (see Supplementary Note 4). In Fig. 1e we plot the addition energy *E*_add_ for increasing hole number. A clear increase in the addition energy is observed for *N* = 2 and *N* = 6, which suggests the orbital shell is full for the second and sixth holes.

Further evidence for orbital shell filling is given by the stair-like disappearance of charge transition signals, which is highlighted by the dashed horizontal white lines in Fig. 1d. Along each vertical charge transition line the measured signal decreases as *V*_C_ is made more positive. As *V*_C_ becomes more positive the tunnel barrier becomes more opaque, and subsequently the tunnel rate from the dot to the reservoir *Γ* decreases. The charge sensor transition is no longer visible when *Γ* < 2*f*_pulse_, as shown schematically in Fig. 1f. When a hole in the dot occupies a higher energy orbital shell, its wavefunction span increases, which increases the tunnel rate. Hence, the charge sensor transition signals should lose visibility at more positive *V*_C_ for holes in higher orbitals. A statistical analysis of the visibility of the charge transition lines (see Supplementary Note 2) shows that *N* = (1, 2), (3, 4, 5, 6), and (7, 8) charge transitions become unmeasurable with the pulsed gate technique at almost the same *V*_C_ (dashed lines in Fig. 1d). These groupings suggest that these holes fill the same orbital state, with similar tunnel rates in the same orbital level.

These observations of shifts in addition energy and tunnel rate suggest that the first two holes fill into the first orbital, and the next four holes fill into the second orbital. This shell filling is consistent with the Fock–Darwin orbital structure for a 2D parabolically confined quantum dot^28,29^. Beyond *N* = 6 the observed orbital filling departs from the so-called 2D magic numbers, which may reflect a loss of circular symmetry of the parabolic confinement for higher hole occupation, since the higher orbital hole wavefunctions are more sensitive to a non-circular confinement profile. Other possibilities include many-body effects^30^, which further reduce the energy spacing between different shells.

### Characterizing the spin filling sequence

**Fig. 2:**
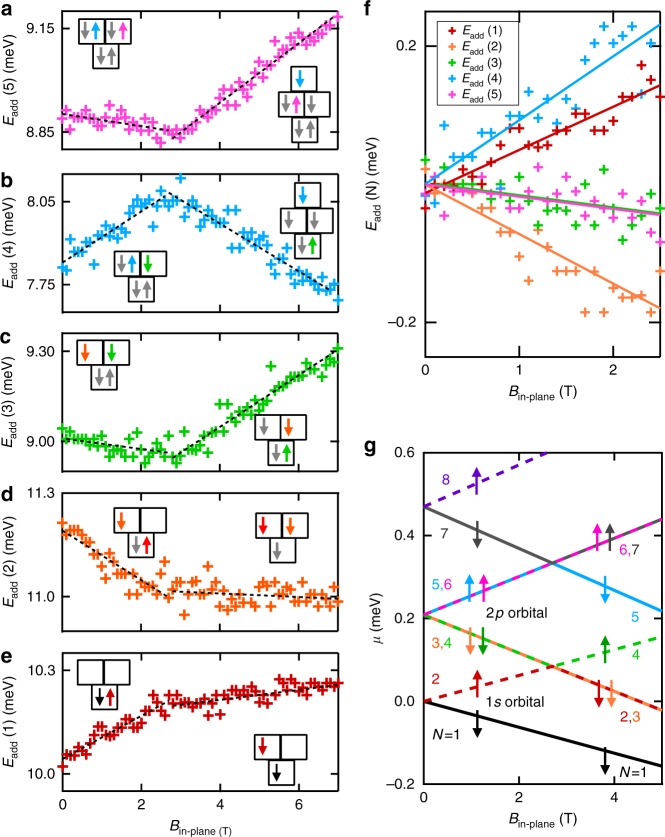
Spin filling sequence and orbital structure. **a**–**e** Additional energy for the first six holes as a function of in-plane magnetic field. The *N*th addition energy, *E*_add_(*N*) = *μ*_*N*+1_ − *μ*_*N*_ is calculated by measuring the spacing between consecutive Coulomb peaks, that is, between the (*N* + 1)th and *N*th peaks. The black dashed lines are a linear fit to the raw data over the region |*B*| < 2.7 T (low field) and |*B*| > 2.7 T (high field). The left and right inset shows the inferred ground state spin filling for the low and high magnetic field regions, respectively. Here, the vertical stacking of the squares represents the orbital sturcture. Each colored arrow corresponds the *N*th hole (as depicted by the colored text in **g**). Since *E*_add_(*N*) measures the spacing between the *μ*_*N*+1_ and *μ*_*N*_, only the two states relevant to the respective addition energy measurement are shown in color, all other states are gray. **f** The addition energies of **a**–**e** plotted over the low field region with data offset to clarify the three distinct slopes, positive, negative, and close to zero. Solid lines are the same least-squares fit to the data in **a**–**e**. The difference in the slope of *E*_add_(1) and *E*_add_(4) is due to the different orbital g*-factors. **g** Model of the hole orbital shell structure and energy levels for the first eight holes (ignoring Coulomb charging energy), extracted directly from the data presented in **a**–**e**. Each line corresponds to the hole charge occupations in **a**–**e**, and the color of each line corresponds to the color of the numbers on the left and right of the figure. See Methods section for additional experimental parameters

To understand the spin structure of the hole quantum dot, we study the magnetic field dependence of the addition energy of the hole dot for *N* = 1 to 6 holes. In Fig. 2a–e we show the addition energy *μ*_*N*+1_ − *μ*_*N*_ for the first six holes as a function of in-plane magnetic field *B*. The slope of the *N*th addition energy *E*_add_ with respect to *B* depends on the relative spin orientation of the (*N* + 1)th and *N*th hole, with three distinct possibilities:

1 $$\frac{{{\mathrm{d}}E_{{\mathrm{add}}}}}{{{\mathrm{d}}B}} = \begin{array}{*{20}{l}} { + g^ \ast \mu _{\mathrm{B}}} \hfill & { \downarrow \uparrow } \hfill \\ { - g^ \ast \mu _{\mathrm{B}}} \hfill & { \uparrow \downarrow } \hfill \\ 0 \hfill & { \uparrow \uparrow {\kern 1pt} {\mathrm{or}}{\kern 1pt} \downarrow \downarrow } \hfill \end{array},$$

where the first and second arrow depicts the spin filling sequence of the (*N* + 1)th and *N*th holes, respectively. We refer to |*B*| < 2.7 T as the low field region, and |*B*| > 2.7 T as the high field region. In both the low and high field region of Fig. 2a–e, the slope d*E*_add_/d*B* is either positive, negative, or close to zero, as shown by the dashed lines. Figure 2f shows that the slope of the addition energy for the first six holes in the low field region takes one of three distinct values, consistent with Eq. (1).

In Fig. 2a–e we observe a change in slope at 2.7 T. This change in slope suggests a change in the spin filling sequence, which is a result of magnetic field-induced orbital level crossings. Knowing the hole number and the relative spin orientations, we can build up the spin shell filling structure in both the low and high field regimes, as indicated in the left and right insets of Fig. 2a–e (see Supplementary Note 5). We have extracted the orbital effective *g*-factors g* from Fig. 2f to be $g_{1s}^ \ast = 1.1$ and $g_{2p}^ \ast = 1.4$. The effective *g*-factor for the orbital occupied by the 7th hole (*N* = 7) is $g_{N = 7}^ \ast = 1.6$, and can be extracted from the slope of Fig. 2b for *B* larger than 2.7 T. The orbital dependence of g* provides further evidence for the observed orbital structure and is due to the strong spin–orbit coupling of holes^31,32^.

We now discuss the spin filling sequence in detail, beginning with the low field spin filling. The first and second holes form a Pauli spin pair in a twofold degenerate orbital, labeled 1*s*. The third and fourth holes fill the 2*p*_*x*_ and 2*p*_*y*_ states with spins parallel to each other. The fifth and sixth holes fill the 2*p*_*x*_ and 2*p*_*y*_ states with spins parallel to each other, but opposite to the third and fourth holes. We are confident in the assignment of the second orbital as a fourfold degenerate 2*p* orbital consistent with the expected spectrum of a symmetric 2D quantum dot. We can rule out the possibility that the apparent fourfold degeneracy arises from an accidental degeneracy of adjacent orbitals by considering together the additional energy measurements of Fig. 1e, the evidence of corresponding tunnel rate shifts of Fig. 1d, and the distinct effective *g*-factors extracted from Fig. 2a–e.

In Fig. 2g we present the hole orbital spectrum extracted directly from the measurements of *E*_add_ in Fig. 2a–e. Whereas previous studies of silicon hole quantum dots typically show alternating spin filling^33–36^, the spin shell filling observed here is consistent with studies of 2D electrons in high-quality GaAs quantum dots^27,30^. A key result of this work is the observation of consecutive filling of holes with the same spin orientation (↓↓ and ↑↑), which occurs in the 2*p* orbital. Further, the degeneracy of the 2*p*_*x*_ and 2*p*_*y*_ orbital levels at *B* = 0 demonstrates that the quantum dot has remarkably circular confinement. The results in Fig. 2g provide a clear demonstration of the orbital shell spin structure of the first eight holes in a surface-gated silicon quantum dot. In particular, we highlight the observation that holes have spin polarized filling of the 2*p* orbital, analogous to Hund’s first rule of orbital shell filling in atomic physics.

We now discuss the spin filling sequence for |*B*| ≥ 2.7 T. The change in slope of *E*_add_(1), *E*_add_(2), and *E*_add_(3) at *B* = 2.7 T can be attributed to a magnetic field-induced crossing of the 1*s* and 2*p* orbitals, as shown in Fig. 2g). By calculating the Zeeman energy at the 1*s* and 2*p* crossing, we determine the singlet–triplet energy spacing *E*_ST_ for the two-hole dot is ~0.2 meV.

The change in slope around 2.7 T for *E*_add_(4) and *E*_add_(5) can be attributed to a crossing between the 2*p* orbital and the next highest orbital level. The next highest orbital level above the 2*p* orbital is twofold degenerate and is occupied by the 7th and 8th holes as depicted by the solid gray and dashed purple lines in Fig. 2 (see Supplementary Note 5). For circular 2D confinement, the orbital level above 2*p* is expected to be sixfold degenerate. We suspect that the twofold degenerate orbital above the 2*p* orbital may result from a loss of circular symmetry of the dot due to for higher hole occupations, or many-body effects^30^.

### Excited state spectroscopy

**Fig. 3:**
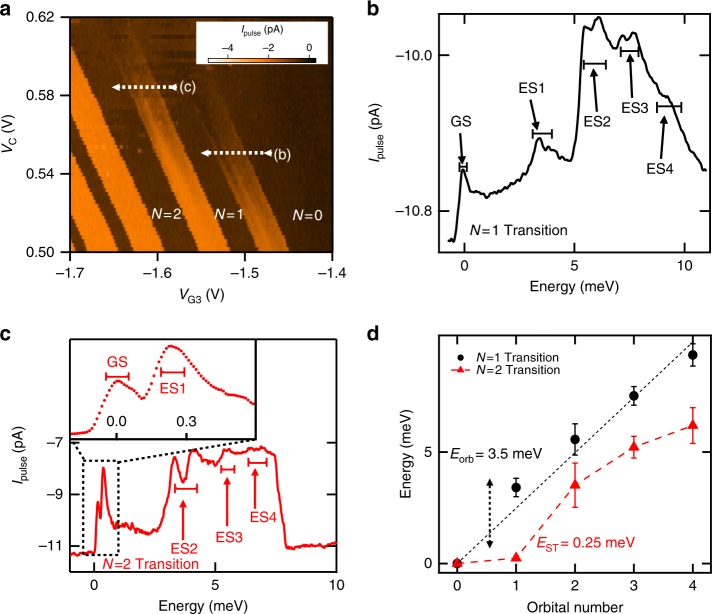
Excited state spectroscopy. **a** Charge stability diagram for *V*_pulse_ = 40 mV and *f*_pulse_ = 333 Hz. The white dashed lines (labeled (b), (c)) correspond to the region where high resoloution cuts are taken to obtain the data in **b** and **c**, respectively. **b** Measurement of *I*_pulse_ over the *N* = 0 → 1 Coulomb peak. The *x*-axis has been converted to energy using the lever arm (Supplementary Note 4). The ground state (GS) and excited states (ES1–4) for the one-hole system are labeled, with the brackets indicating the experimentally determined width of each peak. Additional structure is observed for ES2 and ES3, see text. **c** Same as **b** for the *N* = 1 → 2 Coulomb peak. The inset demonstrates that the ground state and first excited state are resolvable. Each dot represents a single data point. **d** Plot of the extracted excited state energies for the one-hole (black) and two-hole (red) system. The black dashed line is a straight line fit to the *N* = 1 data, highlighting the linear dependence of the excited state energies on the orbital number. The red dashed line is a trend line connecting all the measured *N* = 2 data points (red triangles). The black and red error bars correspond to the width of the black and red brackets in **b**, **c** respectively. See Methods section for additional experimental parameters

To further study the orbital shell structure, and the nature of the confinement potential, we examined the excited state spectrum of the quantum dot. Figure 3a shows the charge stability diagram when *V*_pulse_ is increased to 40 mV. Increasing *V*_pulse_ broadens the charge transition window, allowing single-hole tunneling to occur via either the ground state or an excited state. The excited state spectrum can be resolved by observing the additional structure of *I*_pulse_ within broadened charge transition lines.

Figure 3b shows the excited state spectrum for the dot with single-hole occupation. This spectrum is obtained from a high-resolution cut of *I*_pulse_ vs. *V*_G3_ along the dashed white line labeled (b) in Fig. 3a. The *x*-axis in Fig. 3b is converted to energy using the lever arm (see Supplementary Note 4) and the ground state is referenced as the zero in energy. Peaks in Fig. 3b correspond to the single-hole tunneling into different orbital states in the unoccupied quantum dot (0 → 1 transition).

The extracted orbital energies are plotted as black circles in Fig. 3d, and show a linear dependence on orbital number. This linearity suggests that the confinement of the dot is parabolic. We note that additional structure can be observed in *I*_pulse_ for the second excited state (ES2) and the third excited state (ES3) in Fig. 3b. This additional structure is likely due to orbital splitting resulting from ellipticity of the dot for higher energy orbitals, consistent with results in Fig. 1d.

We now estimate the expected excited state energy scales in order to compare with the experiment. The quantum dot radius was estimated to be ~27 nm, by approximating the dot as a parallel plate capacitor and using the charging energy of 12 meV for the one-hole to two-hole charge occupation (see Supplementary Note 6 and Supplementary Eq. (3)). A dot radius of 27 nm is smaller than previous silicon MOS hole quantum dots operating in the few hole regime^22,23,37^. The expected orbital spacing for a 2D artificial atom with 27 nm radius is ~3 meV (see Supplementary Note 6 and Supplementary Eq. (6)), which is consistent with the measured orbital spectrum in Fig. 3d.

Finally, we investigated the energy spectrum of the two-hole quantum dot. We can determine the strength of hole–hole interactions within the quantum dot by comparing the two-hole energy spectrum with the one-hole energy spectrum. Figure 3c shows the excited state spectrum for the two-hole quantum dot, which is a cut along the dashed white line labeled (c) in Fig. 3a. A key feature of the two-hole dot is that the first excited state is now only 0.25 meV above the ground state (inset of Fig. 3c), while the separation between excited states remains comparable to the *N* = 1 transition excited state energy separation of ~3 meV. The reduction in the spacing between the ground state and first orbital state (3.5 meV for the one-hole system, and 0.25 meV for the two-hole system) results from the additional Coulomb interaction energy when one hole already occupies the lowest energy orbital. The observation of a 0.25 meV excited state spacing for the two-hole dot is consistent with the 0.2 meV Zeeman energy required to induce a singlet–triplet ground state transition in Fig. 2e. Based on the change in first orbital energy spacing, we estimate that the hole interaction energy is ~90% of the orbital energy. The measured hole–hole interaction energy is much larger than the electron-electron interaction energy measured in GaAs^26^ and silicon^38^ lateral quantum dot devices. Large hole–hole interaction energies (compared to the orbital energy) have also been observed in laterally defined GaAs hole quantum dots^39^.

In summary, we have demonstrated a silicon MOS-based quantum dot operating in the last hole regime. The orbital level spacing and the spin filling suggests that the confinement potential is close to parabolic. We have extracted the ground state spin filling from *N* = 1 to 8 holes. These results show that holes in a planar circular quantum dot follow the standard 2D artificial atom spectrum observed in high-quality GaAs electron-based devices^27,30^. We emphasize that this spectrum is achieved due to the tight confinement provided by the surface gate structure of the device. Finally, we observe polarized spin filling and determine that strong hole–hole interactions affect the two-hole energy spectrum. These results highlight the unique physics of 2D hole artificial atoms, and clearly demonstrate that spin properties and energy scales are very different to nanowire and electron artificial atoms^3,33–35^.

## Methods

### The sample

The device studied in this work was fabricated using a high resistivity natural (001) silicon substrate. The P+ ohmic regions are prepared by boron diffusion. A 5.9 nm gate dielectric (SiO_2_) is grown by dry oxidation in the active region of the device. The gate pattern is fabricated using multilayer Al-Al_2_O_3_ gate stack technology^37^. The final stage is a forming gas (95% N_2_, 5% H_2_) anneal to reduce Si/SiO_2_ interface disorder and enhance low temperature performance. All measurements were performed in a dilution fridge with a base temperature below 30 mK.

When operating the device, the reservoir top gate is negatively biased to accumulate a 2D hole system at the Si/SiO_2_ interface below. The quantum dot is defined by positively biasing gates G1, G2, G4, and the C-gate. G3 acts as the dot plunger gate and is operated in the negatively biased regime. It is possible to operate this device in the double dot regime down to the (0, 0) charge state, using gate G2 as the second dot’s plunger gate. Additionally, in the double dot regime we can observe interdot tunneling. See Supplementary Note 3 for more information on the tunability of this device.

It is expected that the hole density is approximately 10^12^ cm^−2^, and the thickness of the 2D layer is of the order of 5 nm. This thickness is less than the in-plane magnetic length for all data presented in this work. Therefore, we consider in-plane magnetic field-induced orbital effects to be negligible. For calculations we take the effective hole mass to be 0.21*m*_0_ based on Luttinger parameters^40^.

The specific gate voltages for each data set are given here. For Fig. 1c–e, *V*_R_ = −3.50 V,*V*_G1_ = −3.50 V, *V*_G2_ = −1.01 V, and *V*_G4_ = 0 V. For Fig. 2a–f, *V*_R_ = −3.50 V, *V*_C_ = +0.55 V, *V*_G1_ = −3.50 V, *V*_G2_ = −0.73 V, and *V*_G4_ = −0.10 V. Note that the change in voltage applied to G2 in Fig. 2 accounts for the small shift in measured addition energy between Figs. 1e and 2a–e. For Fig. 3a, *V*_R_ = −3.50 V, *V*_G1_ = −3.50 V, *V*_G2_ = −1.01 V, and *V*_G4_ = 0 V. For Fig. 3b, *V*_R_ = −3.50 V, *V*_C_ = +0.55 V, *V*_G1_ = −3.50 V, *V*_G2_ = −1.01 V, and *V*_G4_ = 0 V. For Fig. 3c, *V*_R_ = −3.50 V, *V*_C_ = +0.585 V, *V*_G1_ = −3.50 V, *V*_C_ = −1.01 V, and *V*_G4_ = 0 V.

### Charge sensor

We present measurements of the charge sensor signal, which are obtained using the pulse-bias charge sensing method described in the main text. Further technical details of this technique can be found in refs ^26,38^. In order to maximize the sensitivity of the charge sensor, we use a feedback loop on the sensor gate^41^. This feedback is achieved using a linear correction, which adjusts the sensor’s left barrier gate voltage, *V*_SLB_, to compensate for changes in *I*_sensor_ due to sweeping *V*_G3_ and *V*_C_. We note that the charge transitions signals using *I*_pulse_ in Fig. 1d are sensitive to the dot to reservoir tunnel rate; hence, we have also confirmed the charge occupation by simultaneously measuring the sensor conductance using *I*_sensor_, which is not sensitive to the tunnel rate. See the Supplementary Note 3 for further details.

### Square pulse parameters

The 333 Hz square pulse with 50% duty cycle is provided using a Tabor wx1284 arbitrary wave generator. The pulse is added to the DC bias on G3 using a room temperature passive adder. The rise and fall time of the square pulse is measured to be 100 ns, which is limited by the AC + DC adder. Refer to Supplementary Note 3 for more details. For measurements of the tunnel rate see Supplementary Fig. 3a–c.

### Magneto-spectroscopy

In order to infer the spin configuration of the dot for different hole occupations, we measure the spin state of all additional holes relative to the previous hole . The *N* = 1 spin ground state is assigned as down. The relative spin orientation of the first six holes can be inferred from the data presented in Fig. 2a–e, and is well described by the orbital model presented in Fig. 2g. Further discussion regarding the spin filling of the 7th and 8th holes is provided in the Supplementary Note 5.

### Pulse-bias spectroscopy

The charge stability diagram shown in Fig. 3a is obtained using the same gate bias configuration as the stability diagram in Fig. 1b, except that the charge transitions are broader due to increased *V*_pulse_ to 40 mV. When sweeping *V*_G3_ over a broadened charge transition signal (as indicated by the horizontal dashed lines in Fig. 3a), *I*_pulse_ initially increases as the ground state is pulsed below the reservoir electrochemical potential *μ*_res_. *I*_pulse_ then decays as *V*_G3_ becomes more negative, since the effective tunnel barrier increases. For sufficiently negative *V*_G3_ additional excited states become accessible for tunneling, which increases the tunnel rate and causes additional spikes in *I*_pulse_.

### Data availability

The datasets generated during and analyzed during the current study are available from the corresponding author on reasonable request.

## Electronic supplementary material

Supplementary Information

## References

[1] Loss D, DiVincenzo DP (1998). Quantum computation with quantum dots. Phys. Rev. A.

[2] Hanson R, Kouwenhoven LP, Petta JR, Tarucha S, Vandersypen LMK (2007). Spins in few-electron quantum dots. Rev. Mod. Phys..

[3] Zwanenburg FA (2013). Silicon quantum electronics. Rev. Mod. Phys..

[4] Veldhorst M (2014). An addressable quantum dot qubit with fault-tolerant control-fidelity. Nat. Nanotech..

[5] Koppens FHL (2006). Driven coherent oscillations of a single electron spin in a quantum dot. Nature.

[6] Koppens FHL, Nowack KC, Vandersypen LMK (2008). Spin echo of a single electron spin in a quantum dot. Phys. Rev. Lett..

[7] Szumniak P, Bednarek S, Partoens B, Peeters FM (2012). Spin–orbit-mediated manipulation of heavy-hole spin qubits in gated semiconductor nanodevices. Phys. Rev. Lett..

[8] Kloeffel C, Trif M, Stano P, Loss D (2013). Circuit qed with hole–spin qubits in Ge/Si nanowire quantum dots. Phys. Rev. B.

[9] Bulaev DV, Loss D (2005). Spin relaxation and anticrossing in quantum dots: Rashba versus dresselhaus spin–orbit coupling. Phys. Rev. B.

[10] Keane ZK (2011). Resistively detected nuclear magnetic resonance in *n*- and *p*-type GaAs quantum point contacts. Nano Lett..

[11] Brunner D (2009). A coherent single-hole spin in a semiconductor. Science.

[12] De Greve K (2011). Ultrafast coherent control and suppressed nuclear feedback of a single quantum dot hole qubit. Nat. Phys..

[13] Maurand R (2016). A CMOS silicon spin qubit. Nat. Commun..

[14] Watzinger, H. et al. Ge hole spin qubit. Preprint at https://arxiv.org/abs/1802.00395 (2018).

[15] Pribiag VS (2013). Electrical control of single hole spins in nanowire quantum dots. Nat. Nanotechnol..

[16] Golovach VN, Borhani M, Loss D (2006). Electric-dipole-induced spin resonance in quantum dots. Phys. Rev. B.

[17] Flindt C, Sørensen AS, Flensberg K (2006). Spin–orbit mediated control of spin qubits. Phys. Rev. Lett..

[18] Nowack KC, Koppens FHL, Nazarov YV, Vandersypen LMK (2007). Coherent control of a single electron spin with electric fields. Science.

[19] Dennis E, Kitaev A, Landahl A, Preskill J (2002). Topological quantum memory. J. Math. Phys..

[20] Jones C (2018). Logical qubit in a linear array of semiconductor quantum dots. Phys. Rev. X.

[21] Li R, Hudson FE, Dzurak AS, Hamilton AR (2015). Pauli spin blockade of heavy holes in a silicon double quantum dot. Nano Lett..

[22] Spruijtenburg PC (2013). Single-hole tunneling through a two-dimensional hole gas in intrinsic silicon. Appl. Phys. Lett..

[23] Yamaoka Y, Iwasaki K, Oda S, Kodera T (2017). Charge sensing and spin-related transport property of p-channel silicon quantum dots. Jpn. J. Appl. Phys..

[24] Betz AC, Gonzalez-Zalba MF, Podd G, Ferguson AJ (2014). Ambipolar quantum dots in intrinsic silicon. Appl. Phys. Lett..

[25] Veldhorst M (2015). A two-qubit logic gate in silicon. Nature.

[26] Elzerman JM, Hanson R, Willems van Beveren LH, Vandersypen LMK, Kouwenhoven LP (2004). Excited-state spectroscopy on a nearly closed quantum dot via charge detection. Appl. Phys. Lett..

[27] Tarucha S, Austing DG, Honda T, Van der Hage RJ, Kouwenhoven LP (1996). Shell filling and spin effects in a few electron quantum dot. Phys. Rev. Lett..

[28] Fock V (1928). Bemerkung zur quantelung des harmonischen oszillators im magnetfeld. Z. Phys. A.

[29] Darwin CG (1931). The diamagnetism of the free electron. Math. Proc. Camb. Philos. Soc..

[30] Ciorga M (2000). Addition spectrum of a lateral dot from coulomb and spin-blockade spectroscopy. Phys. Rev. B.

[31] Nenashev AV, Dvurechenskii AV, Zinovieva AF (2003). Wave functions and *g* factor of holes in Ge/Si quantum dots. Phys. Rev. B.

[32] Pryor CE, Flatté ME (2006). Landé *g* factors and orbital momentum quenching in semiconductor quantum dots. Phys. Rev. Lett..

[33] Roddaro S (2008). Spin states of holes in Ge/Si nanowire quantum dots. Phys. Rev. Lett..

[34] Zwanenburg FA, van Rijmenam CEWM, Fang Y, Lieber CM, Kouwenhoven LP (2009). Spin states of the first four holes in a silicon nanowire quantum dot. Nano Lett..

[35] Hu Y, Kuemmeth F, Lieber CM, Marcus CM (2012). Hole spin relaxation in Ge-Si core-shell nanowire qubits. Nat. Nanotechnol..

[36] Brauns M, Ridderbos J, Li A, Bakkers EPAM, Zwanenburg FA (2016). Electric-field dependent *g*-factor anisotropy in Ge-Si core-shell nanowire quantum dots. Phys. Rev. B.

[37] Li R, Hudson FE, Dzurak AS, Hamilton AR (2013). Single hole transport in a silicon metal-oxide-semiconductor quantum dot. Appl. Phys. Lett..

[38] Yang CH (2012). Orbital and valley state spectra of a few-electron silicon quantum dot. Phys. Rev. B.

[39] Bogan A (2017). Consequences of spin–orbit coupling at the single hole level: spin-flip tunneling and the anisotropic *g* factor. Phys. Rev. Lett..

[40] Winkler, R. *Spin–Orbit Coupling Effects in Two-dimensional Electron and Hole Systems*, Springer Tracts in Modern Physics, 191 (Springer, Berlin, 2003).

[41] Yang CH, Lim WH, Zwanenburg FA, Dzurak AS (2011). Dynamically controlled charge sensing of a few-electron silicon quantum dot. AIP Adv..

