# Supplementary material for: Spin and orbital structure of the first six holes in a silicon metal-oxide-semiconductor quantum dot
Source: Nat Commun. 2018 Aug 14;9:3255. doi: 10.1038/s41467-018-05700-9 (PMC6092405; doi:10.1038/s41467-018-05700-9)
Supplement: Supplementary file 1 — Supplementary Information [file 41467_2018_5700_MOESM1_ESM.pdf]

Supplementary Information: Spin and orbital structure of the first six holes in a silicon MOS quantum dot

Liles, S. D. *et al.*

Supplementary Note 1 - Confirming absolute charge occupation of the dot, indpendent of tunnel rate

The absolute hole occupation N presented in the main text is inferred from measurements of I_{pulse} in Figure 1d. Since I_{pulse} is sensitive to the dot tunnel rate Γ[1], it is possible that additional charge transitions are present, but are not detected by the pulsed gate measurements since the tunnel time $1/\Gamma$ is longer than the pulse length $1/2f_{\text{pulse}}$. In this section of the Supporting Information we show that the sensor conductance measures the absolute charge occupation and confirms the charge transitions observed in I_{pulse}. Further, we show that the sensor conductance is sensitive to charge transitions, even when the tunnel rate is significantly smaller than f_{pulse}.

In Supplementary Figure 1a we present a charge stability diagram obtained by measuring I_{pulse} while sweeping V_{G3} and V_{C} over a larger range than presented in Figure 1d of the main text. In addition to measuring I_{pulse} at $f_{\text{pulse}} = 333Hz$, we simultaneously monitor the sensor conductance by adding a 50μV 77Hz excitation to the sensor source ohmic contact and measuring the AC sensor signal I_{sensor} at 77Hz using a standard lock-in measurement. The sensor conductance was primarily monitored in order keep the sensor at approximately constant conductance using a feedback loop. I_{pulse} is most sensitive to charge transitions when the sensor conductance is tuned to the region of highest transconductance. However, since the sensor is capacitively coupled to all nearby gates, the sensor conductance will drift away from the most sensitive region over large range sweeps of V_{G3} and V_{C}. In order to maintain maximum sensitivity we employ a dynamic feedback method[2]. The dynamic feedback is achieved using a linear correction, which adjusts the sensor barrier gate V_{SLB} in order to compensate for changes in I_{sensor} due to sweeping V_{G3} and V_{C}.

In Supplementary Figure 1b we show the measurement of I_{sensor} obtained simultaneously to the measurement shown in Supplementary Figure 1a. We observe charge transitions in I_{sensor}, which correspond to the I_{pulse} charge transition signals in Supplementary Figure 1a. The charge transition signals in I_{sensor} are weak since the dynamic feedback maintains I_{sensor} approximately constant over linear sweeps of V_{G3} and V_{C}. However, at certain V_{G3} the dot charge occupation changes by one hole. The electric charge of an additional hole causes a spike in I_{sensor}, which is not initially compensated for by the feedback on V_{SLB}. This is observed as a clear step in the I_{sensor}, indicated by diagonal black lines in Supplementary Figure 1b. The charge transitions observed in I_{sensor} correspond exactly to the transitions observed in the pulsed gate measurements I_{pulse} shown in Supplementary Figure 1a.

In Supplementary Figure 1a horizontal dashed lines show the values of V_{C} where $\Gamma \sim 2f_{\text{pulse}}$ (see main text). In Supplementary Figure 1b horizontal lines are drawn at the same values of V_{C} and V_{G3} as in Supplementary Figure 1a. Each charge transition signal in I_{sensor} continues well beyond the V_{C} at which the corresponding I_{pulse} charge transition is no longer visible. This demonstrates that I_{sensor} is sensitive to charge transitions that have very low tunnel rates. We see no additional charge transitions in I_{sensor} beyond the last transition providing further evidence that the device is operating down to the last hole.

Supplementary Note 2 - Charge Transition visibility and V_{C} groupings

In Figure 1d of the manuscript horizontal white lines are draw to highlight the stair-like disappearance of the charge transition visibility. The specific V_{C} at which the horizontal lines are drawn has been determined by extracting the value of V_{C} at which the charge transition signal in I_{pulse} is no longer distinguishable from the back-ground signal.

In order to determine a value of I_{pulse} that characterizes the loss of visibility, we take a histogram of the data presented in the charge stability diagram of Figure 1d) of the main text. In Supplementary Figure 2a) we show this histogram with I_{pulse} on the x-axis and number of counts on the y-axis . The histogram shows three clear distributions. The largest distribution is the background signal, centered at I_{pulse} approximately 0pA. The two other distributions, centered at I_{pulse} = -1.1pA and I_{pulse} = -0.7pA make up the rising and falling edges of the detectable charge transition signal respectively. The blue arrow marks the range of I_{pulse} = (-0.15,-0.22)pA, which corresponds to the range of I_{pulse} readings where a charge transition is detected. To demonstrate this, in Supplementary Figure 2b) we plot the charge

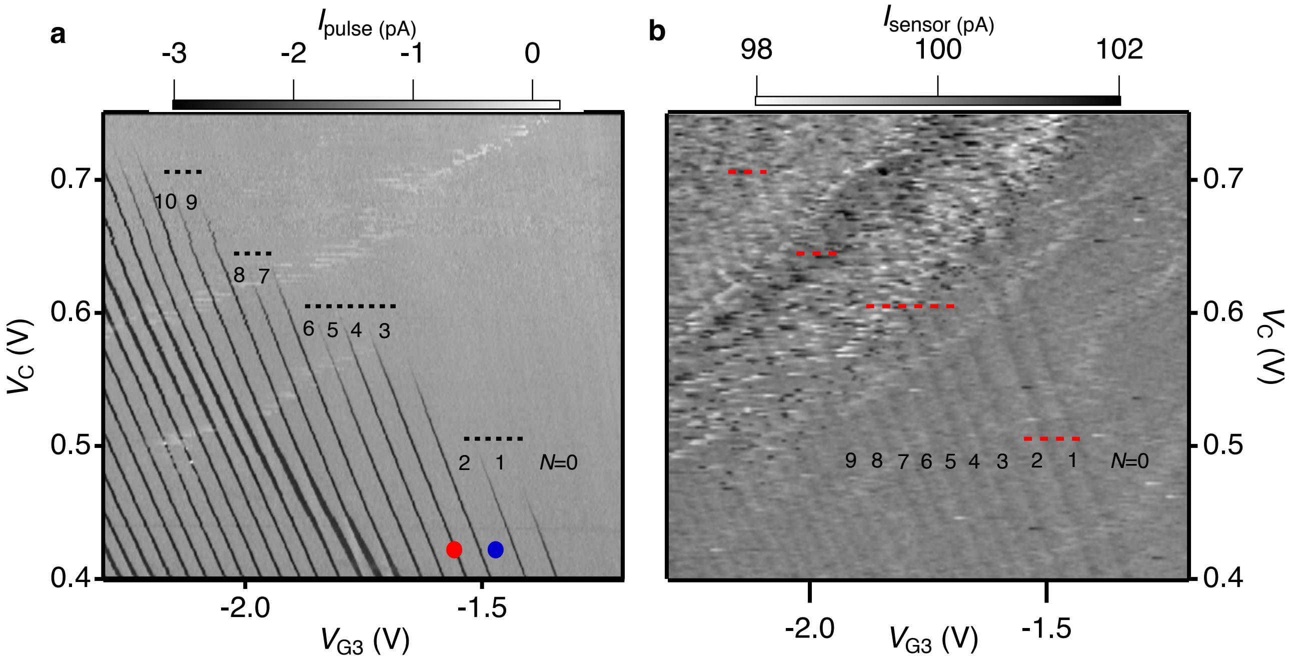

Supplementary Figure 1: **Supplementary Stability Diagram.** (**a**) I_{pulse} charge stability diagram. The horizontal black lines highlight the disappearence of the charge transition signals, which occurs as V_C become more positive causing the tunnel rate for to become much less than $f_{\text{pulse}} = 333\text{Hz}$. The red and blue circle represent the device configuration for the red and blue data in Supplementary Figure 3a. (**b**) Measurument of I_{sensor} obtained simultaneously to the data presented in (a). Horizontal red lines are drawn at the same V_C and V_{G3} as the black dashed lines in **a**, highlighting that the I_{sensor} charge transitions are visible well beyond the V_C when the I_{pulse} charge transition signals are no longer visible. For both Supplementary Figures **a** and **b** the experimental parmeters are the same as Figure 1d of the main text, excpet V_{D2} = -1.0. The difference in V_{D2} explains the difference in the position of the black dashed lines between this Supplementary Figure and the white dashed lines in Figure 1d of the main text.

stability diagram with a binary color scale, with black for values of I_{pulse} indicated by the blue arrow in Supplementary Figure 2a) (transition) and white for any value of I_{pulse} outside this range (background).

Based on the distribution of I_{pulse} we are able to extract a value of V_C at which the signal is no longer visible. The error bars are determined from the width of the distributions, and the observed decay in I_{pulse}. Supplementary Figure 2c) shows the extracted values of V_C for the charge stability diagram in Supplementary Figure 2b). The horizontal black lines in 2c) correspond to the horizontal lines drawn in Figure 1d of the main text. The distinct groupings are highlighted by the red brackets on the right of Supplementary Figure 2c). There is no overlap between the distinct groupings, demonstrating the effect of orbital shell filling.

Supplementary Note 3 - Tuning the dot-to-reservoir tunnel rate and operation of the device as a double quantum dot

Supplementary Figure 3a shows that the dot tunnel rate can be observed in the time resolved sensor DC source-drain signal. The y-axis in Supplementary Figure 3a is the average DC sensor signal $\langle I_{\text{DC}} \rangle$, which is plotted as a function of time. To obtain $\langle I_{\text{DC}} \rangle$ we perform 1024 single shot measurements over a 3ms square wave, which is applied to G3. $\langle I_{\text{DC}} \rangle$ is the time resolved average of all 1024 single shot measurements. The blue data in Supplementary Figure 3a is a measurement of $\langle I_{\text{DC}} \rangle$ when the dot is far away from a charge transition (indicated by the blue circle in Supplementary Figure 1a). $\langle I_{\text{DC}} \rangle$ follows the shape of the square wave due to capacitive coupling of the sensor conductance to the load and empty phase of the square pulse. The red data in Supplementary Figure 3a is the same measurement of $\langle I_{\text{DC}} \rangle$, however the dot is now tuned to be at a charge transition (indicated by the red circle in Supplementary Figure 1b.

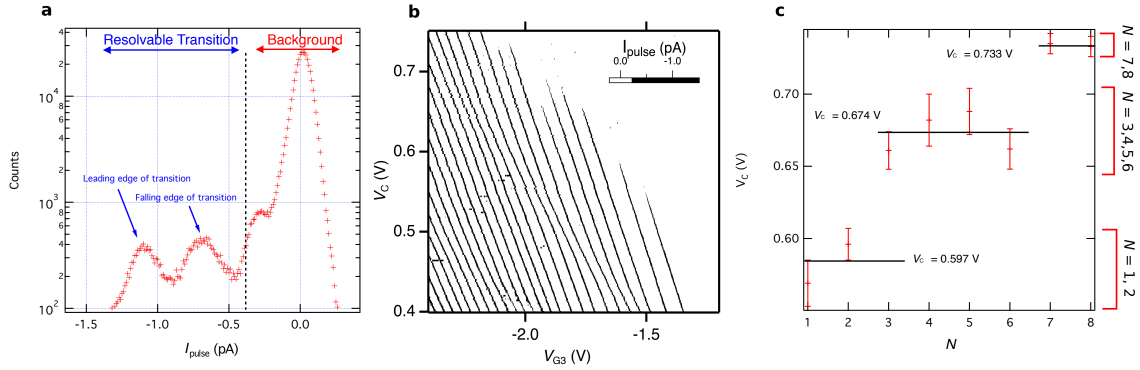

Supplementary Figure 2: **Charge transition histogram and groupings.** **(a)** Histogram of the data presented in the charge stability diagram of Figure 1d of the main text. The x-axis shows bin centres for values of measured I_{pulse}, while the y-axis shows the number of counts within the bins. The blue arrow highlights the values of I_{pulse} that corespond to charge transition signals, while the red arrow highlights the values of I_{pulse} that corespond to background signal. The peak centred at approximately 0pA is the background signal. The two peaks at -1.1pA and -0.7pA can be identified from the data as the rising and falling edges of the pulse-detected charge transition. **(b)** The charge stability diagram from the main text of the manuscript Figure 1d) is shown with with binary colour scale. For the charge transition signal, $I_{\text{pulse}} <$ -0.22pA, data is shown as black, while for the background single, $I_{\text{pulse}} >$ -0.22pA, data is shown as white. This allows the extraction of the background signal and easy identification of the loss of visiablity of each charge transition. **(c)** The extracted values of V_{C} (with uncertainty) at which each transition loses visability. There is a clear overlap of the N = (1,2) the N=(3,4,5,6) and N = (7,8). The horizontal black lines highlight this overlap, and are draw at the same respective V_{C} as Figure 1d in the main text. The red brackets on the right side highlight the groupings. The error bars are the deviation in the mean V_{C} value at which the charge transition is no longer visable.

In the load phase, where the square pulse is in the negative phase, $\langle I_{\text{DC}} \rangle$ initially increases as the sensor conductance follows the capacitive coupling. However, when a hole tunnels onto the dot, the additional positive charge causes an exponential decay in $\langle I_{\text{DC}} \rangle$. In the empty phase, where the square pulse is in the positive phase, the reverse of this process occurs. $\langle I_{\text{DC}} \rangle$ exponentially decays due to holes tunneling out from the dot, reducing the positive charge and returning $\langle I_{\text{DC}} \rangle$ to the non-transition value. The width of the exponential decay in $\langle I_{\text{DC}} \rangle$ results from the average tunnel time of holes over the 1024 single shot measurements.

In Supplementary Figure 3b we demonstrate that the tunnel rate can be easily tuned by presenting measurements of $\langle I_{\text{DC}} \rangle$ at three different V_{G2}. As V_{G2} is made more negative the tunnel barrier height between the dot and reservoir is decreased, causing a decrease in the dot to reservoir tunnel time. We extract a measurement of the tunnel time by fitting an exponential function to the decay in $\langle I_{\text{DC}} \rangle$ associated with from charge tunneling on or off the dot. In Supplementary Figure 3c we plot the extracted tunnel time for V_{G2} = -0.715 V, -0.725 V and -0.735 V, which is found to be 0.37±0.07 ms, 0.13±0.01 ms and 0.11±0.05 ms respectively.

When V_{G2} is made sufficiently negative G2 no longer behaves as a tunnel barrier, but begins to act as an accumulation gate. In Supplementary Figure 3d we show a measurement of I_{pulse} as a function of V_{G2} and V_{G3}. We observe a honeycomb pattern consistent with a double dot charge stability diagram. The absolute hole occupation (N_{G2},N_{G3}), of the double dot can be assigned since we see no additional charge transitions in I_{pulse} or I_{sensor} beyond the region labeled (0,0). The magnitude of the I_{pulse} signal for the G2 dot charge transitions is less than the magnitude of the I_{pulse} signal for G3 dot charge transitions. This is because G2 is further from the sensor, causing the G2 dot to have a weaker capacitive coupling to the sensor. Finally, in Supplementary Figure 3e we show the (0,1) to (1,0) charge transition region. We observed a weak signal in I_{pulse} along the axis between the (0,1) and (1,0) region. This weak signal results from inter-dot charge transitions induced by the square wave (interdot transitions can be seen for many transitions in Supplementary Figure 3d.

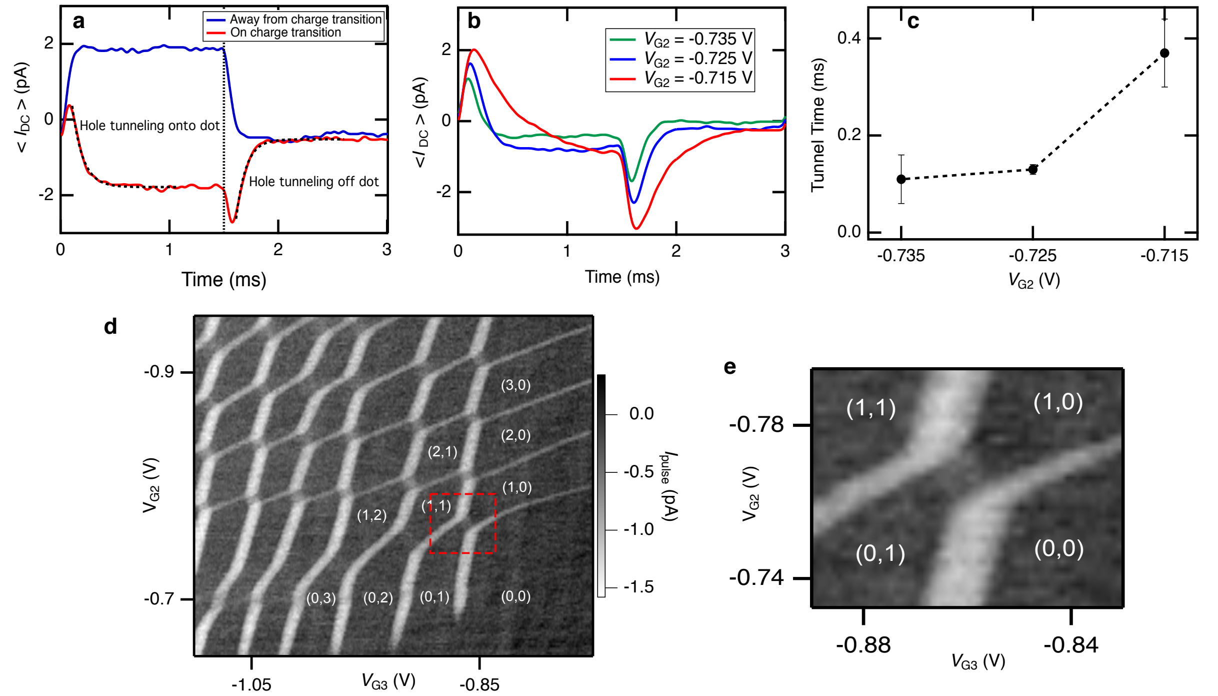

Supplementary Figure 3: **Tunability of the device** (**a**) $\langle I_{\rm DC}\rangle$ measured as a function of time while a 3ms square pulse is applied to G3 ($V_{\rm pulse} = 3$mV). The vertical dotted line separates the x-axis into the load phase (0-1.5ms) of the square pulse and the empty phase (1.5-3ms)of the square pulse. The blue and red lines are measurements when the device is in the configuration shown by the blue and red circles in Supplementary Figure 1a. The black dashed lines are an exponential fit to the decay of the $\langle I_{\rm DC}\rangle$ signal. (**b**) $\langle I_{\rm DC}\rangle$ for progresively more negative V_{G2}. (**c**) The hole tunnel time as extracted from an exponential fit to the decay in $\langle I_{\rm DC}\rangle$ for each trace in **b**. the error bars are extracted from the standard deviation of the fit to the exponential decay (600 data points). (**d**) $I_{\rm pulse}$ double dot charge stability diagram for $V_{\rm G3}$ and V_{G2} ($V_{\rm pulse} = 10$mV, $f_{\rm pulse} = 333$Hz). White text shows the hole occupation (N_{G2},$N_{\rm G3}$). (**e**) Zoom-in of the (0,1) to (1,0) charge transiution in **d**.

Supplementary Note 4 - Lever arm and hole temperature calculation

The lever arm α is a linear coefficient allowing a change in gate voltage to be converted to a change in the electrochemical potential $\Delta E = \alpha \Delta V_{\rm G3}$. Typically in quantum dots, the lever arm α is determined by varying the source drain bias, and measuring so called coulomb diamonds in single dot transport[3]. However, since the device described in this letter is a single lead quantum dot, transport measurements through the single dot are not possible. In order to calculate the lever arm we first tune $V_{\rm G3}$ so that the single dot under G3 is on a charge transition (in this case we use the 2nd to 3rd hole transition). We then apply an 8mV sawtooth wave to G3 causing the hole occupation of the dot to shift from two holes to three holes over the sawtooth sweep. The average DC sensor current $\langle I_{\rm DC}\rangle$ will shift from high to low as the sawtooth sweeps the dot electro-chemical potential across the reservoir chemical potential. The width in $V_{\rm G3}$ of the shift from high to low $\langle I_{\rm DC}\rangle$ is determined by the Fermi distribution of hole states in the reservoir[4,5].

In Supplementary Figure 4a we show $\langle I_{\rm DC}\rangle$ for two different fridge mixing chamber temperatures $T_{\rm MXC}$. The width of the transition in $\langle I_{\rm DC}\rangle$ is sensitive to the thermal broadening of the reservoir distribution of hole states. We fit $\langle I_{\rm DC}\rangle$ to the function

$$\langle I_{\rm DC}\rangle = \frac{I_{\text{peak-to-peak}}}{1+\exp[\frac{(V_{\rm G3}-V_0)\alpha e}{k_{\rm B}T}]} + I_{\rm offset} = \frac{a}{1+\exp[\frac{V_{\rm G3}-b}{c}]} + d \qquad (1)$$

where a, b, c, d are fitting parameters. The smooth lines in Supplementary Figure 4a show the fit to the raw data. By comparing the fitting function to the Fermi-Dirac distribution we determine that the parameter

$$c = \frac{k_{\rm B}}{\alpha e}\sqrt{T_0^2 + T_{\rm MXC}^2} \qquad (2)$$

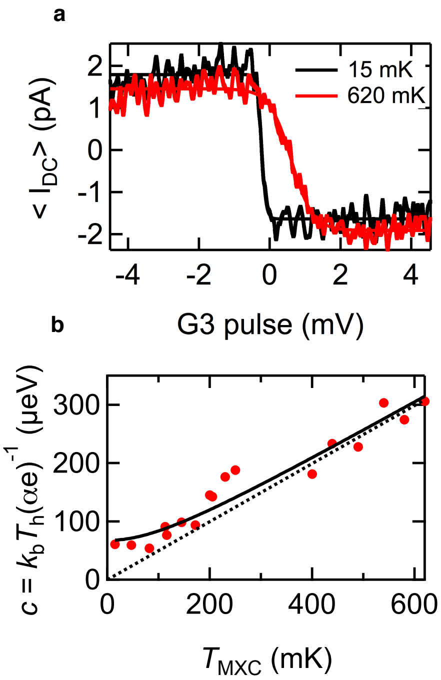

Supplementary Figure 4: **Lever arm calculation.** (**a**) $\langle I_{\mathrm{DC}} \rangle$ resulting from an 8mV sawtooth wave applied to G3 at fridge remperatures of 15mK and 620mK. (**b**) Fitting parameter c (Equation 1) as a function of fridge temperatue T_{MXC}. Typical values for the remaining fitting parameters of Equation 1 are approximately constant at a = 3.6pA, b = 3.2x10^{-1}mV, $d = -1.8$pA. T_{h} is the hole temperature temperature, which depends on the hole bath temperature T_0 and the mixing chamber temperature T_{MXC} (Equation 2). The solid black line is a fit to Equation 2, giving T_0 = 136mK, and α = 0.174 eV/V. The dashed black line is a least squares fit for the data where T_{MXC} is much larger than 136mK. The resulting slope of the dashed black line gives α = 0.174 eV/V.

where T_0 is the constant hole bath temperature, and T_{MXC} is the dilution fridge mixing chamber temperature. Therefore, it is possible to extract the lever arm by measuring the temperature dependence of the fitting parameter c.

In Supplementary Figure 4b we plot the fitting parameter c as a function of the fridge mixing chamber temperature T_{MXC}. On the range of 15mK to 130mK the fitting parameter c is unaffected by changes in the mixing chamber temperature T_{MXC}. Above 130mK the fitting parameter c increases linearly with T_{MXC}, as shown by the black line in Supplementary Figure 4b. We extract the minimum hole bath temperature to be 100mK. For T_{MXC} above 100mK we infer that the hole bath temperature T_{h} is equal to T_{MXC}. The G3 lever arm α_{G3} is calculated to be 0.174 eV/V from fitting the parameter c as a function of T_{MXC} to Supplementary Equation 2. The G3 lever arm α_{G3} was calculated for the two to three hole charge transition. We use α_{G3} = 0.174 eV/V for all analysis presented in the main text. We note that the lever arm is expected to be dependent on the hole occupation due to additional screening and changes in the dot size. However, the linearity of charge transitions for the first 12 holes, shown in Supplementary Figure 1a, allows us to confidently infer that the lever arm is approximately constant at 0.174 eV/V for at least the first 12 holes.

Supplementary Note 5 - Spin filling of the 7th and 8th holes

Here, we discus details regarding the assignment of the spin filling and orbital degeneracy for the 7th and 8th holes. Although the addition energy for the 7th and 8th holes is not directly measured, it is possible to determine the spin filling and orbital degeneracy based on the high field slope of $E_{\mathrm{add}}(4)$ and $E_{\mathrm{add}}(5)$ in Figures 2a and 2b of the main text. In Figure 2b, for $B>$2.7T $E_{\mathrm{add}}(4)$ decreases with increasing B, demonstrating that the 5th hole ground state is spin down for $B>$2.7T. Addition energy measurements in Figure 1e show that the second orbital is full for six holes. Thus, for five holes there are no remaining spin down states available in the second orbital. Therefore we conclude that the change in spin filling sequence at B=2.7T of the 5th hole is due to Zeeman induced crossing between the 2p orbital and the

next highest orbital. We conclude that the 7th and 8th holes occupy a two-fold degenerate orbital. This two-fold degeneracy is supported by the observation that the 5th and 6th holes fill with opposite spin for at high field, as opposed to the 6th and 7th holes, which fill with spin aligned to the high field due to the four-fold degeneracy of the $2p$ orbital.

Supplementary Note 6 - Calculation of the dot size and First excited state energy

In order to estimate the dot diameter we approximate the dot-gate system as a parallel plate capacitor and use the measured N=1 to N=2 addition energy, $E_{\text{add}} = 12$ meV shown in Figure 1e of the main text. The parallel plate capacitance is given by,

$$C = \frac{\epsilon_r \epsilon_0 A}{d} \tag{3}$$

where A is the equivalent capacitor area, and d is the oxide thickness. The capacitance is equated to the standard single dot charging energy,

$$E_{\text{C}} = E_{\text{add}} = \frac{C}{e} \tag{4}$$

Taking the oxide thickness, d, to be 5.9nm and relative permitivity, ϵ_r to be 3.9 we find the equivalent parallel plate capacitor Area, A, to be 2.28x10^{-15}m^2. For a circular dot this gives a radius of 27nm.

The approximate excited state energy is calculated by modeling the dot as an isotropic two dimensional harmonic oscillator. We use the ground state Fock-Darwin wavefunction given by

$$\psi_0(r) = (\frac{m^*\omega}{2\pi\hbar})^{\frac{1}{4}} e^{\frac{-m^*\omega r^2}{4\hbar}} \tag{5}$$

where r is the radial dimension, and m^* is the effective mass of heavy holes in silicon. We take the in effective heavy hole mass to be m^*=$0.21m_0$ using Luttinger parameters[6]. To evaluate the excited state energy, $\hbar\omega$, we solve the probability density function, $\langle\psi|\,|\psi^*\rangle$, and set the point of inflection to be half the calculated dot radius.

$$\frac{d^2}{dr^2}\psi^2 = 0 \tag{6}$$

The value of r at the point of inflexion is given by $r = \sqrt{\frac{\hbar}{m^*\omega}}$. Equating $r = \frac{27nm}{2}$ gives $\omega = 4 \times 10^{12}\text{s}^{-1}$, corresponding to an excited state energy of $\hbar\omega = 3$meV.

References

1. Elzerman, J. M., Hanson, R., Willems van Beveren, L. H. Vandersypen, L. M. K., & Kouwenhoven, L. P, Excited-state spectroscopy on a nearly closed quantum dot via charge detection, *Applied physics letters*, **84**(23), 4617-4619 (2004).

2. Yang, C. H., Lim, W. H., Zwanenburg, F. A., & Dzurak, A. S. Dynamically controlled charge sensing of a few-electron silicon quantum dot, *AIP Advances*, **1**(4), 042111 (2011).

3. Kouwenhoven, L. P., Austing, D. G., & Tarucha, S. Few-electron quantum dots, *Reports on Progress in Physics*, **64**(6), 701-736 (2001).

4. Morello, A. et al. Single-shot readout of an electron spin in silicon, *Nature*, **467**(7316), 687-691 (2010).

5. Prance, J. R. et al. Single-shot measurement of triplet-singlet relaxation in a Si/SiGe double quantum dot, *Physical Review Letters*, **108**(4), 046808 (2012).

6. Lawaetz, P. Valence-band paramters in cubic semiconductors, *Phys. Rev. B*, **4**(10), 3460-3467 (1971).
